# Validation of reference genes for normalization of qPCR gene expression data from *Coffea spp*. hypocotyls inoculated with *Colletotrichum kahawae*

**DOI:** 10.1186/1756-0500-6-388

**Published:** 2013-09-28

**Authors:** Andreia Figueiredo, Andreia Loureiro, Dora Batista, Filipa Monteiro, Vítor Várzea, Maria Salomé Pais, Elijah K Gichuru, Maria Céu Silva

**Affiliations:** 1 Plant Systems Biology Lab, Center of Biodiversity, Functional & Integrative Genomics (BioFIG), Science Faculty of Lisbon University, Lisbon 1749-016, Portugal; 2 CIFC-Biotrop/IICT-Instituto de Investigação Científica Tropical, Quinta do Marquês, Oeiras 2784-505, Portugal; 3 Coffee Research Foundation (CRF), P.O. Box 4–00232, Ruiru, Kenya

## Abstract

**Background:**

Coffee production in Africa represents a significant share of the total export revenues and influences the lives of millions of people, yet severe socio-economic repercussions are annually felt in result of the overall losses caused by the coffee berry disease (CBD). This quarantine disease is caused by the fungus *Colletotrichum kahawae* Waller and Bridge, which remains one of the most devastating threats to *Coffea arabica* production in Africa at high altitude, and its dispersal to Latin America and Asia represents a serious concern. Understanding the molecular genetic basis of coffee resistance to this disease is of high priority to support breeding strategies. Selection and validation of suitable reference genes presenting stable expression in the system studied is the first step to engage studies of gene expression profiling.

**Results:**

In this study, a set of ten genes (*S24, 14-3-3, RPL7*, *GAPDH, UBQ9, VATP16*, *SAND, UQCC, IDE* and *β-Tub9*) was evaluated to identify reference genes during the first hours of interaction (12, 48 and 72 hpi) between resistant and susceptible coffee genotypes and *C. kahawae.* Three analyses were done for the selection of these genes considering the entire dataset and the two genotypes (resistant and susceptible), separately. The three statistical methods applied GeNorm, NormFinder, and BestKeeper, allowed identifying *IDE* as one of the most stable genes for all datasets analysed, and in contrast *GADPH* and *UBQ9* as the least stable ones. In addition, the expression of two defense-related transcripts, encoding for a receptor like kinase and a pathogenesis related protein 10, were used to validate the reference genes selected.

**Conclusion:**

Taken together, our results provide guidelines for reference gene(s) selection towards a more accurate and widespread use of qPCR to study the interaction between *Coffea spp.* and *C. kahawae*.

## Background

*Coffea arabica* L. production in Africa can be seriously affected by coffee berry disease (CBD), caused by the hemibiotrophic fungus *Colletotrichum kahawae* JM [1]. Despite the fact that several plant organs can be affected, maximum production losses occur when infection takes place in expanding green berries, leading to their premature dropping and mummification [2,3]. Crop damages due to CBD, along with chemical control costs, accounts annually for a loss of US$ 300–500 millions in Arabica coffee production [4].

Since its first report in 1922 in Kenya [5], CBD rapid outbreak throughout Eastern Africa urged the development of breeding programmes in several countries (such as Kenya, Ethiopia and Tanzania) giving rise to the release of several resistant coffee varieties for coffee growers [2,4]. In Kenya, the most relevant example is the hybrid commercial variety Ruiru 11, which was bred for resistance to CBD and coffee leaf rust (*Hemileia vastatrix*) using lines of the coffee cultivar Catimor as resistance sources. Breeding programmes remain the most economical and environmentally sound method for a sustainable coffee production. Presently, with the advances in genomic and transcriptomic resources, breeding for resistance can be supported and thus improved by molecular based-information.

Molecular research on the coffee - *C. kahawae* pathosystem has been lagging behind other plant-*Colletotrichum* spp. interactions although advances on the mechanisms of pathogen infection and host resistance at cellular level were achieved [2,3,6] Deepening the knowledge on the molecular basis governing coffee resistance to *C. kahawae* is thus fundamental to get some insights on the distinctive processes underlying plant resistance response. Monitoring gene differential expression and validating high-throughput RNA sequencing (RNA-seq) data is ideally achieved through quantitative real-time PCR (qPCR) analysis. Regardless of being an extremely powerful technique relative to sensitivity, specificity and broad quantification range, accurate data normalization with a reference gene(s) is an absolute requirement for qPCR correct measurement of gene expression. In this study, we have tested 10 candidate genes for qPCR normalization of gene expression during the first hours of interaction (12, 48 and 72 hpi) with *C. kahawae*. Two coffee genotypes, resistant and susceptible to *C. kahawae* were used. The best combination of reference genes determined for each dataset was used to further assess the expression of a pathogenesis-related protein 10 (*PR10*) and a receptor like kinase (*RLK*) known to be induced during coffee *–* leaf rust interactions [7,8]. Here we provide, for the first time, a set of reference genes suitable for gene expression studies in both resistant and susceptible coffee genotypes to *C. kahawae*.

## Results and discussion

Normalization is one of the key factors affecting the accuracy and reliability of quantitative gene expression analysis. Here, we describe an assessment of ten candidate reference genes (RGs) for their use as internal controls in gene expression studies of coffee defence responses against *C. kahawae* in two different genotypes, Caturra as susceptible and Catimor 88 as resistant. Data were analyzed considering the entire dataset and each of the coffee genotypes separately.

### Amplification specificity and efficiency

To examine the expression stability of the candidate RGs selected, transcript levels of the ten candidates were measured by qPCR using gene-specific primer pairs (Table 1). Melting curves of the genes tested were analysed to detect the absence/presence of primer dimer or non-specific PCR products (Additional file 1). For V-Type proton ATPase (*VATP16*), SAND family protein (*SAND*) and Ubiquinol-cytochrome c reductase complex chaperone (*UQCC*), melting curves profiles revealed non-specific amplification and primer dimer formation on the amplicon region (Additional file 1), rendering these genes as unsuitable for further analysis (Table 1). For all remaining genes, melting curve analysis showed no amplification peak in the no-template controls (NTCs) (Additional file 1).

**Table 1:** Candidate reference genes and target genes primer sequences, amplicon length and qPCR analysis

**Gene (coffee**	**Reference**	**Primer sequence**	**Amplicon length (bp)**	**Ta (°C)**	**Tm (°C)**	**PCR efficiency (%)**	**Regression coefficient (R**^**2**^**)**	**Average Cq value**
**source gene)**								
*VATP16* (GT00156.1)*	Gamm et al. [29]	Fw: ATAGAGAGAGAGCCCCCAATTC	150	60	78.63	Discarded (unspecific amplification, primer dimer)		
		Rev: CCGAGGAAGCCAAAAAAAG						
*UQCC* (isotig09120)		Fw: CCTCGGGCTTCATCTCTACTC	156	60	77.21			
		Rev: TCTCCCCAGCATTTTTTGTC						
*SAND* (isotig05620)	Selim et al. [30]	Fw: GATTTGTCTACGAACCCTGCTT	116	60	78.70			
		Rev: GGACCACCAACACCAATAAAC						
*UBQ9* (AF29708)*	Ramiro et al. [7]	Fw: AACATTGAGGGTGGTTCTGTTC	79	60	76.16	92	0.987	24.5 ± 0.95
		Rev: GCAGAAAACCAACTAAGACCTAACAA						
*S24* (SNG-U349723)^a^	Cruz et al. [28]	Fw: GCCCAAATATCGGCTTATCA	92	60	76.60	91	0.996	20.61 ± 1.04
		Rev: TCTTCTTGGCCCTGTTCTTC						
*IDE* (isotig10635)	Borges et al. [12]	Fw: TGATCTAAGCTGGTGGAAAGC	91	55	76.28	92	0.994	24.46 ± 1.30
		Rev: TCAGGTGCATCAGGATGATT						
*β- Tub9* (isotig08544)		Fw: ACCCTCCAGCAAACTGATGA	100	55	77.27	96	0.996	19.40 ± 0.82
		Rev: AGGATGCCACTGCTGATGAT						
*14-3-3* (SGN-U356404)^a^	Barsalobres-Cavallari et al. [18]	Fw: TGTGCTCTTTAGCTTCCAAACG	75	60	73.33	103	0.999	22.76 ± 1.47
		Rev: CTTCACGAGACATATTGTCTTACTCAAA						
*GADPH* (SGN-U347734)^a^		Fw: TTGAAGGGCGGTGCAAA	59	60	75.73	96	0.998	21.42 ± 1.96
		Rev: AACATGGGTGCATCCTTGCT						
*RPL7* (SGN-U351477)^a^	Barsalobres-Cavallari et al. [18]	Fw: CATTCGAGGTATCAATGCTATGCA	66	60	76.48	89	0.999	23.76 ± 1.28
		Rev: TGTCTCAGGCGCAGAAGCT						
**Genes of interest (GOIs)**								
*PR10* (CF589103)*	Ramiro et al. [7]	Fw: GCCACCATCCTTGAAGAGAA	151	55	80.17	99	0.999	19.35 ± 2.28
		Rev: CAACTCTCTGCTTGGCAGTCT						
*RLK* (CF589181)*		Fw: ATGGGAGAAAAGAATGGCAGAAG	189	55	81.15	91	0.998	24.81 ± 2.02
		Rev: GGCCAATTACAGTTTGAAAACACC						

^a^Unigene accession number according to the SOL Genomics Network.

Ta, annealing temperature; Tm, melting temperature; SD, standard deviation. * NCBI accession number or TC TIGR number.

PCR efficiency of each primer pair was calculated through the standard curve method using a pool of all cDNA samples in a ten-fold serial dilution. The amplification efficiency (E) of the reactions ranged from 1.886 (89%) to 2.033 (103%) with the correlation coefficients R^2^ varying from 0.98 to 0.99 (Table 1). To assure that any variation between biological replicates was not related to the treatments but intrinsic to the gene itself, data from the biological replicates was analysed separately by statistical algorithms [9,10].

### Analysis of gene expression stability data

The expression stability of the selected RGs was addressed by three different statistical applets: GeNorm, NormFinder and BestKeeper. The results were analyzed by dividing the data into three different datasets: all samples in the assay (entire dataset), biotic stress applied to the coffee cultivars Catimor 88 and Caturra, separately.

### GeNorm

The geNorm algorithm calculates the gene expression stability measure (M value) for each gene based on its average pairwise expression ratio relative to each of the other genes in the analysis. A gene displaying a high M value presents a high variance in its expression. After, GeNorm estimates the normalization factor (NF) using the geometric mean of expression levels of *n* best reference genes, using a pairwise variation (V) with a cut-off value of 0.15 as threshold [11].

The gene encoding for an Insuline Degrading Enzyme (*IDE*) presented high stability in all datasets analysed (lower M value), however, different combinations of genes showed low M values when samples were analyzed only considering one coffee genotype. Therefore, the gene pairs indicated for expression normalization were *IDE/*14-3-3 protein (*14-3-3*) (Table 2) for the entire dataset, *IDE/*Tubulin beta-9 (*β-tub9*) (Table 3) for the susceptible cultivar Caturra during inoculation time-course and *IDE/*60S ribosomal protein L7 (*RPL7*) (Table 3) for the resistant variety Catimor 88 during inoculation time-course. The orthologous gene *IDE* was defined as a reference gene for common bean hypocotyls inoculated with *Colletotrichum lindemuthianum*[12]. In other studies, this same gene was also determined as the most stable and suitable for validation of subtractive libraries of common bean during compatible and incompatible interactions with the rust fungus *Uromyces appendiculatus*[13]. *IDE* gene encodes for a peptidase that could be related to the cleavage control of proline-rich signalling proteins [14]. We may point out that due to its important role in basic cell processes, this gene could be a good reference gene. Glyceraldehyde-3-phosphate dehydrogenase (*GAPDH*) and Ubiquitin (*UBQ9*) were the least stable genes in both genotypes.

**Table 2:** Reference genes ranking for the entire dataset calculated by the GeNorm, NormFinder and BestKeeper

**Entire dataset**				
**Gene**	GeNorm	Normfinder	Bestkeeper	
	M	SV	CV ± SD	*r*
***IDE***	0.578(1/2)	0.402(1)	2.54 ± 0.62(2)	0.801*
***14-3-3***	0.578(1/2)	0.624(4)	2.61 ± 0.59(4)	0.723*
***RPL7***	0.604(3)	0.542(3)	1.98 ± 0.47(1)	0.701*
***β-Tub9***	0.684(4)	0.478(2)	2.44 ± 0.47(3)	0.720*
***S24***	0.731(5)	0.610(5)	2.79 ± 0.57(5)	0.660*
***UBQ9***	0.785(6)	0.699(6)	2.23 ± 0.54(6)	0.560
***GAPDH***	1.019(7)	1.597(7)	n.a	n.a

SV. stability value; CV, coefficient of variance; SD, standard deviation of Cq value; *r*. Pearson coefficient of correlation; * *p ≤* 0.001. *p*-value associated with the Pearson coefficient of correlation. Ranking order is presented in parenthesis, n.a., not assigned.

**Table 3:** **Reference genes ranking for Catimor 88 and Caturra inoculated with *C. kahawae***

**Biotic stress**	**Catimor 88 (resistant)**				**Caturra (susceptible)**			
**Gene**	GeNorm	NormFinder	BestKeeper		GeNorm	NormFinder	BestKeeper	
	M	SV	CV ± SD	*r*	M	SV	CV ± SD	*r*
***IDE***	0.640(1)	0.505(2)	2.82 ± 0.69(4)	0.790*	0.392(1/2)	0.358(3)	2.09 ± 0.50(2)	0.786*
***14-3-3***	0.669(2)	0.759(4)	2.59 ± 0.58(3)	0.689*	0.518(5)	0.517(5)	2.71 ± 0.61(5)	0.778*
***RPL7***	0.640(3)	0.626(3)	2.09 ± 0.49(1)	0.619*	0.502(4)	0.455(4)	1.86 ± 0.44(1)	0.684*
***β-Tub9***	0.795(4)	0.479(1)	2.34 ± 0.45(2)	0.726*	0.392(1/2)	0.322(1)	2.37 ± 0.46(4)	0.822*
***S24***	0.914(5)	0.807(6)	2.87 ± 0.59(5)	0.543	0.476(3)	0.341(2)	2.34 ± 0.48(3)	0.787*
***UBQ9***	0.870(6)	0.735(5)	1.75 ± 0.43(6)	0.303	0.648(7)	0.649(7)	2.61 ± 0.63(6)	0.642*
***GAPDH***	1.169(7)	1.779(7)	n.a	n.a	0.600(6)	0.580(6)	2.85 ± 0.58(7)	0.778*

SV. stability value; CV, coefficient of variance; SD, standard deviation of Cq value; *r*. Pearson coefficient of correlation; * *p ≤* 0.05, *p*-value associated with the Pearson coefficient of correlation Ranking order is presented in parenthesis, n.a., not assigned.

**Figure 1:**
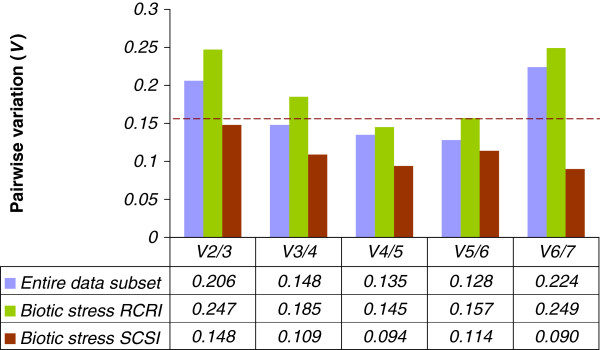
**Pairwise variation (V) of candidate genes as predicted by GeNorm.** The pairwise variation (Vn/Vn + 1) was calculated between the normalization factors NFn and NFn + 1. Each pairwise variation value is compared with a recommended cut-off value 0.15, below which the inclusion of an additional reference gene is not required. RCRI – Catimor 88 inoculated compared to control samples, SCSI – Caturra inoculated compared to control samples.

The analysis carried out by geNorm also enables the determination of the optimal number of reference genes, through the calculation of pairwise variation (V_n_/V_n+1_) between two sequential candidate genes. High values indicate the need for the inclusion of another gene to obtain a reliable normalization factor, which should contain at least two internal controls. Thus, extra reference genes can be included until theV_n_/V_n+1_ value is smaller than a threshold of 0.15 as recommended by Vandesompele et al. [11]. Based on this parameter, the use of three reference genes were set for the entire dataset (V3/4 = 0.148), four reference genes for Catimor 88 (V4/5 = 0.145) and two or three reference genes (V2/3 = 0.148, V3/4 = 0.109) for Caturra hypocotyls inoculated with *C. kahawae* (Figure 1).

### Normfinder

NormFinder is based on a variance estimation approach, which calculates an expression stability value (SV) for each gene analysed. It enables estimation of the overall variation of the RGs, taking into account intra and intergroup variations of the sample set. According to this algorithm, genes with lowest SV will be top ranked [15]. For the entire dataset, *IDE* was considered as the most stable gene (Table 2), while for Caturra and Catimor 88 datasets it appeared in the three top ranked genes, showing in general a high stability as an internal control for coffee hypocotyls inoculated with *C. kahawae*. In addition, the best combination obtained for gene expression analysis of the different genotypes inoculated with *C. kahawae* was β-*Tub9*/40S ribosomal protein S24 (*S24*) for Caturra and *β-Tub9*/*IDE* for Catimor 88 (Table 3). *GAPDH* and *UBQ9* appeared as the least stable genes when analysing the entire dataset and the susceptible genotype Caturra. For the resistant genotype Catimor88, *GAPDH* and *S24* were the most unstable genes.

### Bestkeeper

The BestKeeper tool calculates standard deviation (SD) and the coefficient of variation (CV) based on quantification cycle (Cq) values of all candidate RGs [16]. Reference genes with SD values greater than 1 could be considered as inconsistent and should be excluded. Conversely, genes with the lowest SD value are proposed to be the most stable [16]. BestKeeper estimates the intergene correlation of all reference gene pairs, and the Pearson correlation coefficient (*r*) and the probability (*p*) value are predicted for each correlation. In this study, BestKeeper analysis considered *RPL7* and *β-Tub9* as the most stable genes for the entire dataset, with SD values of 0.47 in both cases (Table 2) with *p* < 0.001. For cultivar Caturra during inoculation time-course *β-Tub9* and *S24* were considered the most stable with SD values of 0.46 and 0.48, respectively, with the best correlations (*r* = 0.882 and 0.787, respectively) and a *p* value of 0.005. For Catimor 88, *IDE* was selected as the most stable with an SD value of 0.69, high correlation (*r* = 0.790) and a p value of 0.005 (Table 3).

Interestingly, despite belonging to important classes of cellular functioning, *GAPDH* and *UBQ9* were indicated by BestKeeper analysis as the least stable expressed genes for the entire dataset and for Catimor 88 under biotic stress, which was consistent with the results obtained from geNorm and NormFinder. *GAPDH* has been widely used as a reference gene in many experimental conditions [17] and is one of the best RGs for measuring gene expression in different *C. arabica* tissues/organs, namely root, stem, leaf, flower and fruit [18]. However previous examples of this gene leading to wrong results have also been reported due to its lack of stability under specific experimental conditions [17,19]. In the present analysis, *GAPDH* was not among the best RGs between experimental groups. One of the possible reasons for those discrepancies may be that *GAPDH* not only acts as a component of the glycolytic pathway but takes part in other processes as well, thus the expression profile of *GAPDH* might fluctuate according to the corresponding experimental conditions. *UBQ9* was also pointed out as a good RG for *C. arabica* leaves inoculated with *Hemileia vastatrix*[7]*.* Yet, for coffee hypocotyls inoculated with *C. kahawae* it was considered one of the least stable genes, which is in accordance with Borges et al. [12] whom pointed out *Ubiquitin* as one of the least stable genes for common bean inoculated with *C. lindemuthianum* in several tissues including hypocotyls. These variations in the expression profiles of genes normally used as internal controls confirm the need for validation of RGs under each specific condition of interest.

A comprehensive ranking analysis considering the three applets (Additional file 2) revealed that, in the specific pathosystem coffee hypocotyls-*C.kahawae*, the two most stable genes, were *IDE* and *β-Tub9* for the resistant genotype and the combination *IDE* + *β-Tub9* or *S24+ β-Tub9* for the susceptible genotype.

### Expression analysis of *PR10* and *RLK*

The coffee *RLK* gene is predicted to encode a receptor-like kinase [20], being some members of this class of proteins involved in resistance signalling pathways [21], while *PR10* seems to be related with the jasmonic acid (JA)-dependent resistance pathway [22] and to accumulate in host cells in incompatible interactions [23,24]. Both *PR10* and *RLK* genes have been described to be induced during coffee infection with *Hemileia vastatrix*[8,20].

The expression of both *PR10* and *RLK* was studied during both coffee compatible and incompatible interactions with *C. kahawae*. Three normalization strategies were followed in order to validate the results obtained by GeNorm, Normfinder and Bestkeeper: 1) using the two best RGs given by the comprehensive ranking, (2) selecting the optimal number of RGs based on GeNorm pairwise variation value (the 0.15 cut-off value was followed) and (3) using the two least stable RGs. Thus, PR10 and RLK expression was normalized in Catimor 88 with a normalization factor (NF) calculated based on the expression of (1) *IDE* and *β-Tub9*, (2) *RPL7*, *IDE*, *14-3-3* and *β-Tub9* and (3) *GADPH* and *UBQ9*. For Caturra, the NF was calculated based on the expression of (1) *IDE* and *β-Tub9* or *S24* and β-Tub9, (2) IDE, S24 and β-Tub9 and (3) GADPH and UBQ9.

**Figure 2:**
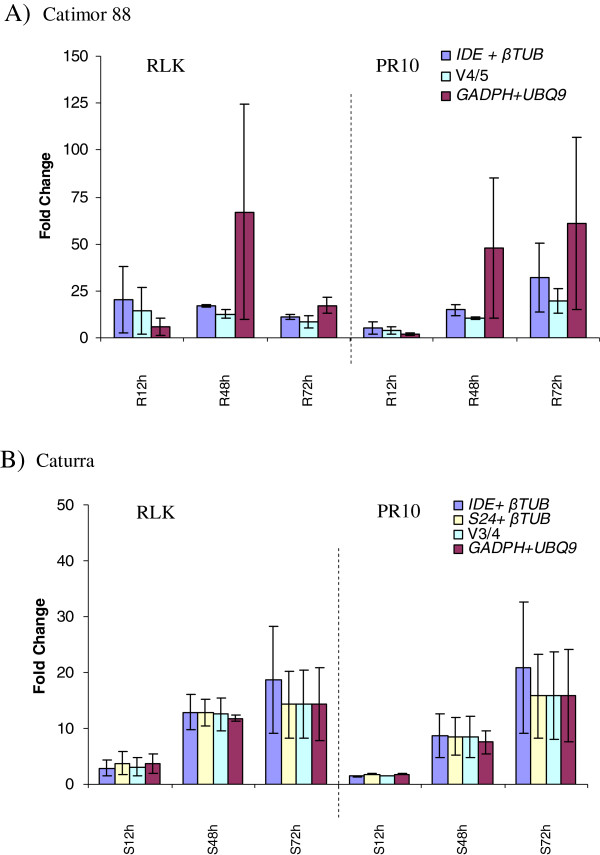
***RLK *and *PR10 *expression in Catimor 88 (A) and Caturra (B) inoculated with *C. kahawae *at 12, 48 and 72hpi.** Three normalization strategies are presented: two best RGs from the comprehensive ranking; GeNorm V value from pairwise variation analysis and the two worst RGs from the comprehensive ranking (*GADPH* and *UBQ9*). Median and SD values of two biological replicates are presented. In Figure 2A, R - resistant genotype; in Figure 2B, S – susceptible genotype.

When comparing the resistant genotype Catimor 88 after inoculation with C. kawahae with control samples, RLK expression increased to reach a maximum at 48hpi and decreased thereafter, while PR10 expression increased during inoculation time-course (Figure 2). Considering the normalization strategies tested, the expression level of both RLK and PR10 did not differ significantly when using the two best RGs from the three applets or the four best RGs given by GeNorm V4/5 value (Figure 1). Using the two best RGs, the expression levels were higher than 11.3 –fold for RLK and 5.5-fold for PR10, decreasing slightly to 8.5-fold for RLK and 4.0-fold for PR10, when data were normalized with the four best RGs from geNorm V = 4/5. In contrast, data normalization with the two more unstable RGs, GADPH and UBQ9, led to significant variable expression levels for both genes analysed, presenting also higher SD values (Figure 2A, Additional file 3), which corroborates the indications from the expression stability analyses that these genes should not be used as RGs for Catimor 88 hypocotyls inoculated with C. kahawae.

For Caturra inoculated with C. kahawae, β-Tub9 appeared as the most stable, followed by both IDE and S24. Thus, normalization with the two best RGs was tested using either a combination of IDE + β-Tub9 or S24+ β-Tub9. When analysing RLK and PR10 expression in comparison to control samples, no significant expression differences were obtain at 12 hpi with the three normalization strategies tested (Figure 2B, Additional file 3). Also, at 48 hpi and 72 hpi, using the two best RGs for normalization (two best genes selected by the statistical applets and also by GeNorm V2/3 value), expression levels and SD values did not varied significantly either with IDE + β-Tub9 or S24+ β-Tub9 (Figure 1). However, when using the combination S24+ β-Tub9, lower SD values were obtained and PR10 and RLK expression levels were very similar to those obtained with the three best RGs given by GeNorm V3/4 value (Additional file 3). It may be considered that despite IDE and S24 shared the second position in the comprehensive ranking of the three statistical applets; S24 is more stable than IDE and thus S24+ β-Tub9 combination should be used for normalization. Interestingly, expression of both genes was not significantly altered when normalizing with the two most unstable genes (GADPH and UBQ9). On the contrary, PR10 and RLK expression levels were slightly smaller or presented higher SD values when normalizing with the three best RGs given by geNorm V = 3/4 (Figure 1). For the susceptible coffee cultivar Caturra inoculated with C. kahawae, despite being considered the most unstable RGs by GeNorm, NormFinder and BestKeeper, GADPH and UBQ9 presented a more stable expression than in the resistant genotype Catimor 88 (Figure 2). These results reflect the need to not only validate reference genes for specific experimental conditions, but also for different genotypes.

A non- parametric test was used to account for any significant differences in the expression of PR10 and RLK calculated with the different normalization strategies (Additional file 4). As no statistically differences were found, we may assume that for an accurate normalization, in the present experimental system, the two most stable RGs suggested by the three applets may be used, i.e. IDE + β-Tub9 for Catimor 88 and S24+ β-Tub9 for Caturra.

## Conclusions

In the present study, we have evaluated the expression stability of 10 candidate reference genes along C. kahawae inoculation time-course of two coffee genotypes showing a susceptible vs resistant response towards this pathogen, aiming to identify a set of stable reference gene(s) for data normalization of gene expression studies. Analysis of expression stability using GeNorm, NormFinder and Bestkeeper revealed that the expression of β-Tub9 and IDE is most stable across all datasets tested. In addition, several normalization strategies were tested under an additional validation step using RLK and PR10, and statistical analysis revealed that for an accurate normalization the two most stable RGs suggested by the three applets should be used and that a different combination of reference genes should be applied for susceptible and resistant genotypes inoculated with C. kahawae. Thus IDE + β-Tub9 should be used for transcript normalization of Catimor 88 hypocotyls inoculated with C. kahawae and S24+ β-Tub9 for transcript normalization of Caturra hypocotyls inoculated with C. kahawae.

Since, up to our knowledge, no reference genes have yet been established for inoculation studies of coffee hypocotyls, we believe that the information provided by our study will greatly facilitate future coffee research and enable sensitive and accurate quantification of gene expression in coffee genotypes showing different degrees of resistance towards C. kahawae.

## Methods

### Inoculation of coffee hypocotyls

Hypocotyls were used as a model material to study CBD in controlled conditions since previous studies have shown a correlation between the pre-selection test on hypocotyls and mature plant resistance in the field (r = 0.73–0.80) [25]. Coffee seeds were sown in greenhouse conditions at temperatures between 16°C and 28°C (average minimum and maximum temperatures respectively), during 8 weeks. Conidia of C. kahawae isolate Que2 (from Kenya) were produced after 7 days at 22°C on extract malt agar [26]. Hypocotyls of cultivars Catimor 88 (from Kenya) and Caturra (CIFC 19/1), resistant and susceptible to the C. kahawae isolate used, were then inoculated according to the technique described [25] with slight modifications. Briefly, hypocotyls were vertically placed on plastic trays containing a wet nylon sponge and sprayed with a conidia suspension (2×10^6^/ml) or with water (mock-inoculated hypocotyls – control samples). After inoculation, trays were covered with plastic bags and and kept in a Phytotron 750 E at 22°C in the dark for 24 h, and then under a photoperiod of 12 hours during the infection time-course.

### Sample collection

According to previous microscopic analysis [27], hypocotyl tissues were sampled at times corresponding to different stages of pathogenesis: i) Conidia germination and appressoria differentiation (in both coffee genotypes) at 12 hpi; ii) fungal penetration and establishment of biotrophic phase (susceptible genotype) or beginning of hypersensitive cell death (HR) and accumulation of phenols (resistant genotype) at 48 hpi; iii) switch to the necrotrophic phase (susceptible genotype) or display of HR and phenols deposition in more that 50% of infection sites (resistant genotype) at 72 hpi. Hypocotyls were thus harvested at 12, 48 and 72 hours post inoculation (hpi). Two independent experiments were conducted and 40 hypocotyls were collected for each coffee genotype (Catimor 88 and Caturra, control and inoculated) at each of the three time-points. Plant material was immediately frozen in liquid nitrogen and stored at 80°C.

### RNA extraction and cDNA synthesis

Total RNA was isolated from hypocotyls with the Spectrum™ Plant Total RNA Kit (Sigma-Aldrich, USA) according to the manufacturer’s instructions. Residual genomic DNA was digested with DNase I (On-Column DNase I Digestion Set, Sigma-Aldrich, USA). RNA purity and concentration were measured at 260/280 nm and 260/230 nm using a spectrophotometer (NanoDrop-1000, Thermo Scientific), while RNA integrity was verified by agarose gel electrophoresis. Genomic DNA (gDNA) contamination was checked by qPCR analysis of a target on the crude RNA [11]. Complementary DNA (cDNA) was synthesized from 2.5 μg of total RNA using RevertAid®H Minus Reverse Transcriptase (Fermentas, Ontario, Canada) anchored with Oligo(dT)_18_ primer (Fermentas, Ontario, Canada), according to manufacturer’s instructions.

### Candidate gene selection and primer design

Ten candidate genes were selected based on their previous reports of suitable qPCR reference genes associated either to Coffea arabica or to biotic stress [7,12,18,28-30]. Five of these genes were previously described as reference genes for C. arabica: S24, 14-3-3, RPL, GAPDH) and UBQ9. The other genes were retrieved from coffee (HDT 832/2) database (unpublished data) as being homologous to grapevine VATP16, SAND, UQCC and homologous to common bean IDE and β*-Tub9*.

Specific primers (Table 1) were designed with Primer Express software version 3.0 (Applied Biosystems, Sourceforge, USA) using the following parameters: amplicon length between 75 and 250 bp; size: 20 ± 2 bp; annealing temperature (Ta) between 55°C and 60°C; GC content: ± 50%.

### Quantitative real time PCR

Quantitative RT-PCR (qPCR) experiments were carried out using Maxima™ SYBR Green qPCR Master Mix (2×) kit (Fermentas, Ontario, Canada) in a StepOne™ Real-Time PCR system (Applied Biosystems, Sourceforge, USA). A final concentration of 2.5 mM MgCl_2_ and 0.2 μM of each primer were used in 25 μL volume reactions, together with cDNA as template. The amplification efficiency of each candidate/target gene was determined using a pool of identical volumes of all cDNA samples. The pool was diluted and used to generate a five-point standard curve based on a ten-fold dilution series. Each standard curve was amplified in two independent qPCR runs and each dilution was run in triplicate. Amplification efficiency (E) was calculated from the slope of the standard curve (E = 10^(−1/a)^ -1) where *a* is the slope of the linear regression model (y = a log(x) + b) fitted over log-transformed data of the input cDNA concentration (y) plotted against Cq values (x).

To investigate candidate reference gene stability, cDNA samples were 10-fold diluted. Thermal cycling for all genes started with a denaturation step at 95°C for 10 min followed by 45 cycles of denaturation at 95°C for 15 s and annealing temperatures (Table 1) for 30 s. Each set of reactions included a negative control with no template. Dissociation curves and agarose gel electrophoresis were used to analyze non-specific PCR products. Two biological replicates and three technical replicates were used for each sample.

### Determination of gene expression stability

The expression stability of each candidate reference gene and the best combination of reference genes were obtained using a pairwise method by GeNorm [11], a model-based method by NormFinder [15] software and the BestKeeper tool [16]. The analysis was performed considering three groups: resistant hypocotyls (Catimor 88) compared to control (mock-inoculated) samples dataset, susceptible hypocotyls (Caturra) compared to control (mock-inoculated) samples dataset and entire dataset. The definition of the optimal number of genes required for normalization was achieved by GeNorm pairwise variation analysis [31]. A comprehensive ranking was established by calculating the arithmetic mean ranking value of each gene using the three applets [32], and each gene was ranked from 1(most stable) to 7 (least stable).

Finally, RefFinder (http://www.leonxie.com/referencegene.php) was used as a verification tool of our results (Additional file 5). RefFinder is a comprehensive tool that integrates the currently available major computational programs (GeNorm, Normfinder, BestKeeper, and the comparative ΔCt method) and based on the rankings from each program assigns an appropriate weight to an individual gene, and calculates the geometric mean of their weights for the overall final ranking [33].

### *PR10* and *RLK* gene expression

The expression of two defense-related genes, a receptor like kinase (*RLK*, CF589181) and a pathogenesis-related protein 10 (*PR10*, CF589103), previously described as being differentially expressed in coffee leaves inoculated with *Hemileia vastatrix*[7,8] was studied in the coffee-*C. kahawae* interaction. Three normalization strategies were tested: 1) using the two top genes given by a comprehensive ranking considering the three methods (GeNorm, NormFinder and BestKeeper); 2) using the optimal number of reference genes selected by the GeNorm V value (pairwise variation analysis, Figure 1) for each condition studied, and 3) using the two most unstable genes considering the comprehensive ranking of the three methods (*GAPDH* and *UBQ9*).

To assess gene expression, relative quantities (RQ) were calculated for both RGs and genes of interest (GOIs) by the formula RQ = E^ΔCq^, where E represents the amplification efficiency (E) for each gene and ΔCq the difference in the Cq from each target sample and calibrator (ΔCq = Cqcalibrator – Cqtarget) [16,34]. A normalization factor calculated as the geometric mean of the relative expression of the RGs selected for each normalization strategy was used to obtain the normalized relative quantities (NRQ) [35].

The statistical significance (p < 0.05) between the three normalization strategies used was determined by the Kruskall-Wallis test using IBM® SPSS® Statistics version 20.0.0 (SPSS Inc., USA) software.

## Competing interests

The authors declare to have no competing interests.

## Authors’ contributions

AF and AL developed the research work. AF participated in the experimental design, performed qPCR experiments, data processing and analysis, participated in data interpretation and wrote the manuscript. AL participated in the experimental design, performed the inoculation experiments, RNA extraction, cDNA synthesis and participated in the manuscript writing. DB participated in the experimental design, plant inoculation experiments, RNA extraction and writing of the manuscript. FM participated in data statistical analysis and interpretation. VV established the fungal inoculum and plant inoculation experiments. EKG supplied the resistant coffee genotype and participated in the study design. MCS participated in the experimental design and inoculation experiments. MSP and MCS were involved in research supervision, data interpretation and critical revising of the manuscript. All authors read and approved the manuscript.

## Supplementary Material

Additional file 1: Figure S1 Primer specificity test through dissociation curve analysis collected from StepOne™ software ver. 2.2.2 (Applied Biosystems). *14-3-3***(A)**, *IDE***(B)**, *RPL7***(C)**, *S24***(D)**, *β-Tub9***(E)**, *GADPH***(F)**, *UBQ9***(G)**, V*ATP16***(H)**, *SAND***(I)**, *UQCC***(J)**, *PR10***(K)** and *RLK***(L)**. Non-template control is indicated by a black arrow. Click here for file

Additional file 2: Table S1 Comprehensive ranking of the candidate genes calculated as the arithmetic mean ranking value of each gene using the three applets. Genes were ranked from the most stable (1) to the least stable (7). Click here for file

Additional file 3: Table S2 *RLK* and *PR10* expression values (fold change) when comparing both inoculated Caturra and Catimor 88 with control samples during inoculation time-course with the different normalization strategies. Click here for file

Additional file 4: Table S3 Test statistics given by the Kruskal-Wallis test on *RLK* and *PR10* expression, comparing the normalization strategies followed. Click here for file

Additional file 5: Table S4 RefFinder final ranking given by the geometric mean of gene position in the ranking from individual tools as geNorm, Normfinder, BestKeeper and the comparative ΔCt methods. Click here for file
