# Supplementary material for: Validation of reference genes for normalization of qPCR gene expression data from Coffea spp. hypocotyls inoculated with Colletotrichum kahawae
Source: BMC Res Notes. 2013 Sep 28;6:388. doi: 10.1186/1756-0500-6-388 (PMC3849654; doi:10.1186/1756-0500-6-388)

**Figure S1-** Primer specificity test through dissociation curve analysis collected from StepOne^TM^ software ver. 2.2.2 (Applied Biosystems).

**A** (*14-3-3*) **B** (*IDE*) **C** (*RPL7*)


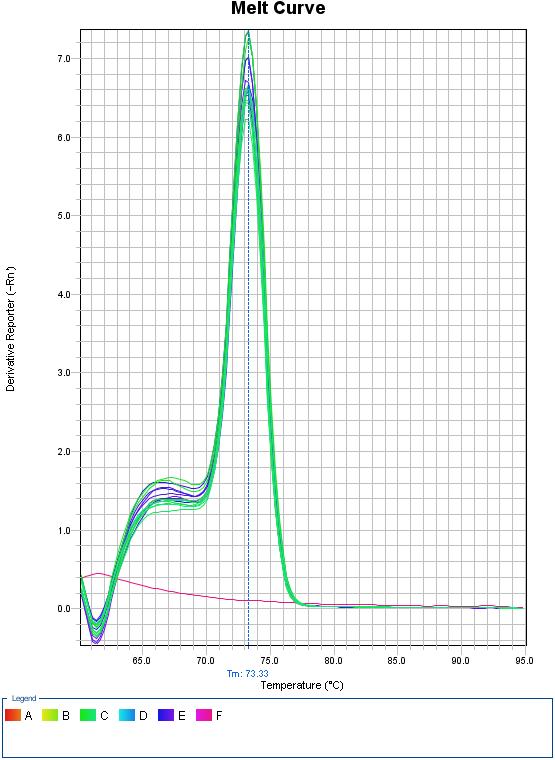

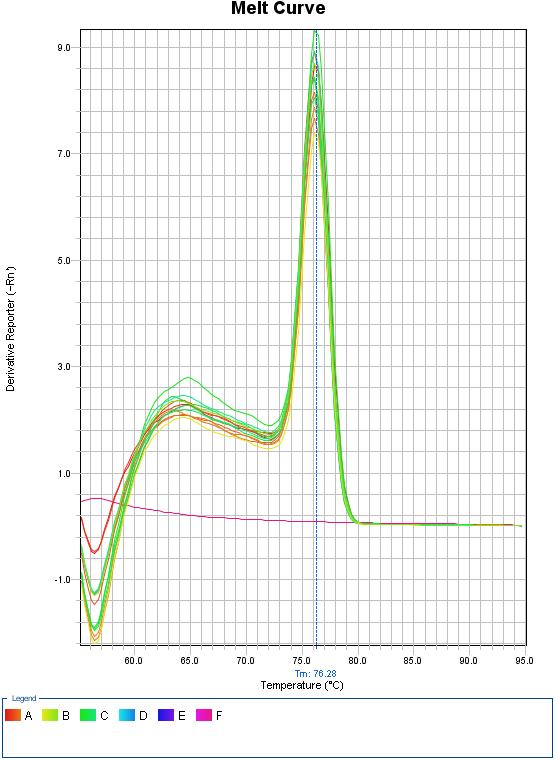

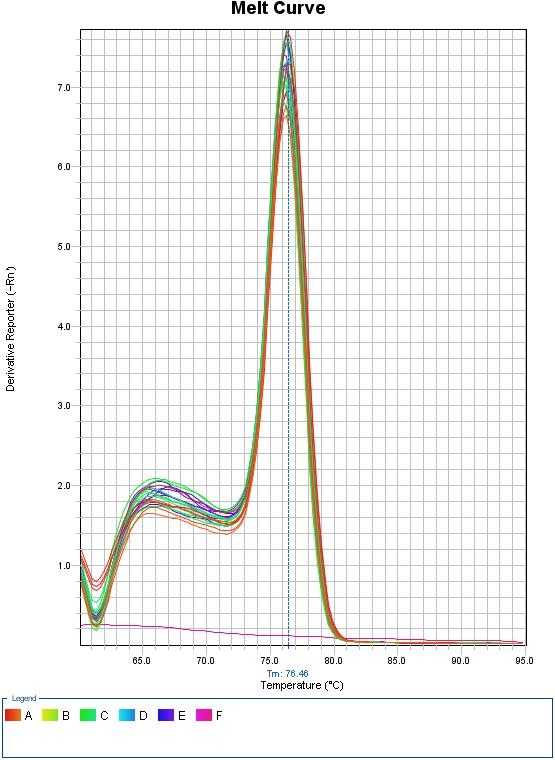


**D** (*S24*) **E** (*β-Tub9*) **F** (*GADPH*)


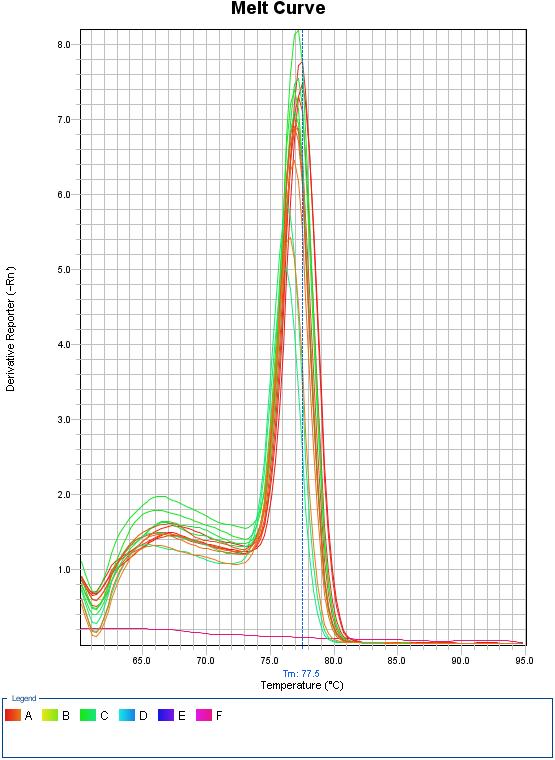

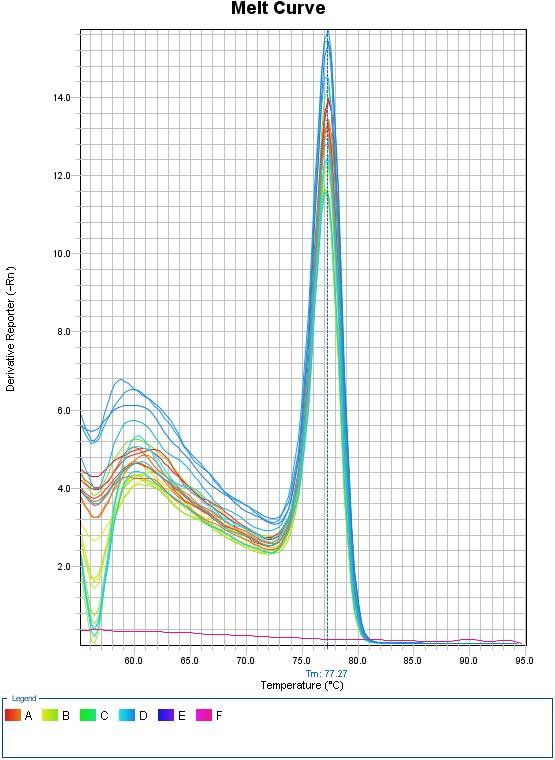

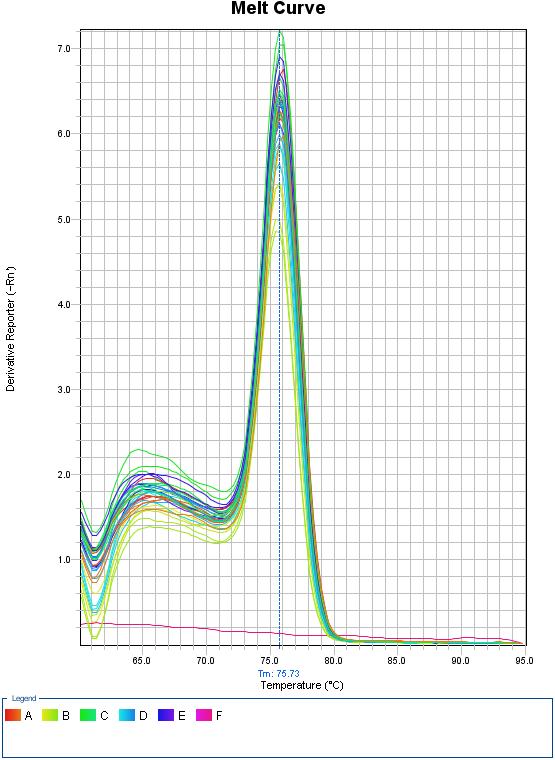


**G** (*UBQ9*) **H** (*VATP16*) **I** (*SAND*)


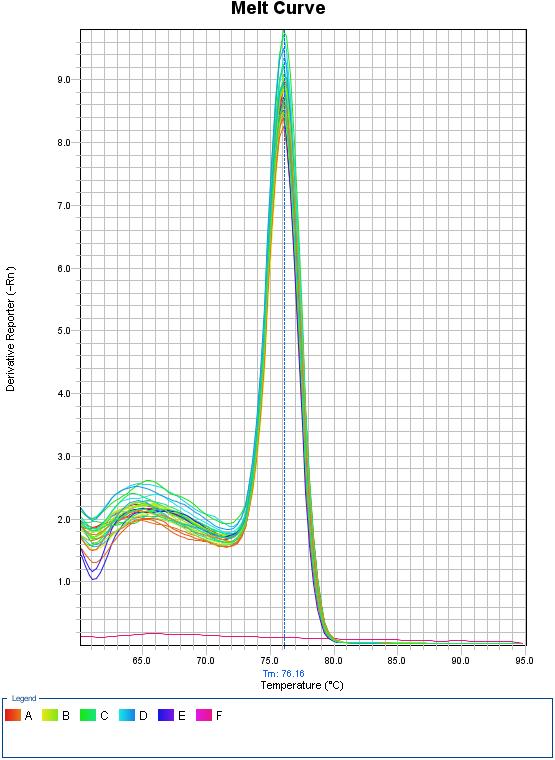

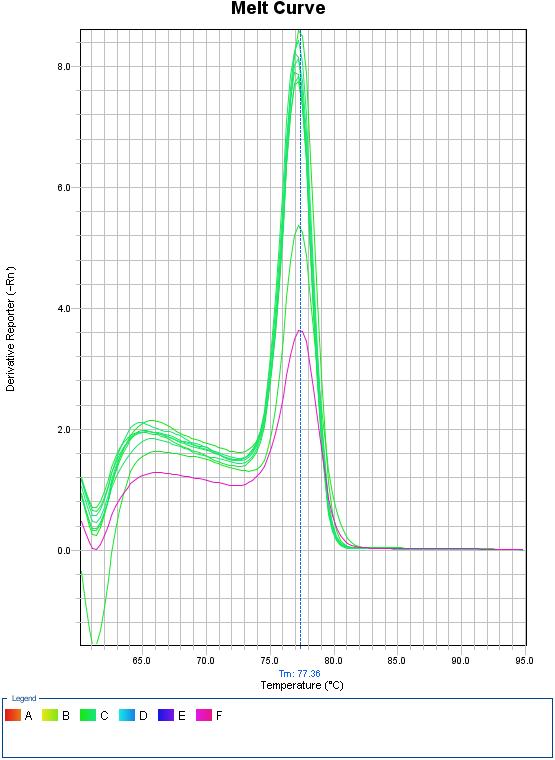

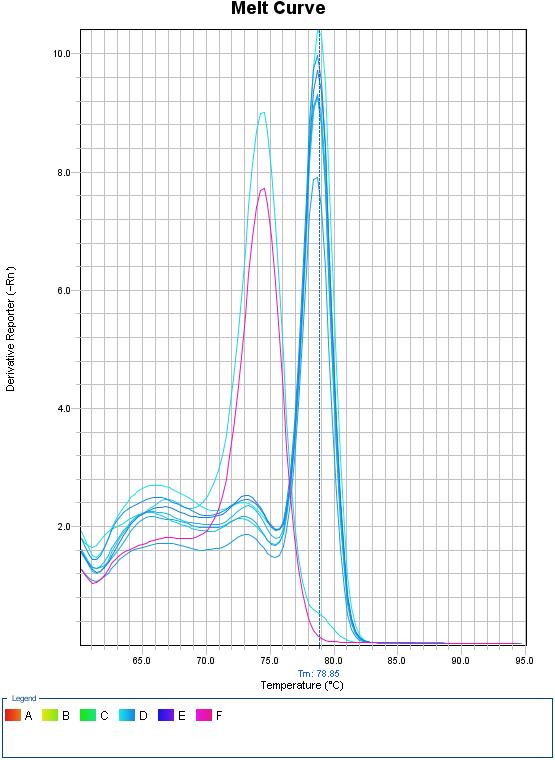


**J** (*UQCC*) **K** (*PR10*) **L** (*RLK*)


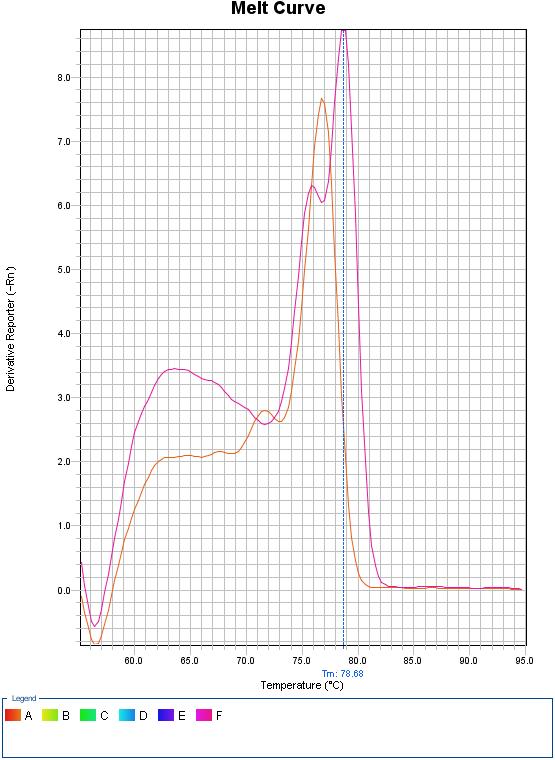

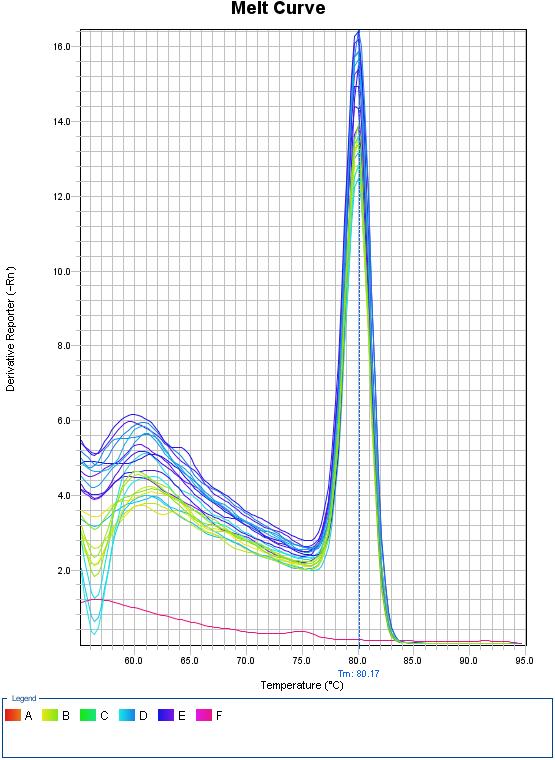

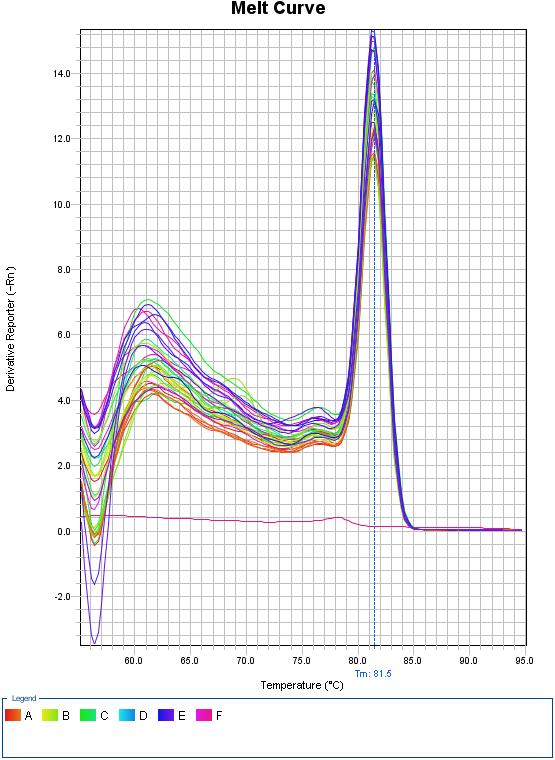

Supplement: Additional file 1: Figure S1 — Primer specificity test through dissociation curve analysis collected from StepOne™ software ver. 2.2.2 (Applied Biosystems). 14-3-3(A), IDE(B), RPL7(C), S24(D), β-Tub9(E), GADPH(F), UBQ9(G), VATP16(H), SAND(I), UQCC(J), PR10(K) and RLK(L). Non-template control is indicated by a black arrow. [file 1756-0500-6-388-S1.doc]
